# Exposure to organochlorine compounds in relation to weight maintenance

**DOI:** 10.21203/rs.3.rs-8097114/v1

**Published:** 2025-12-12

**Authors:** Philippe Grandjean, Alessandra Meddis, Flemming Nielsen, Arne Astrup, Esben Budtz-jørgensen

**Affiliations:** University of Southern Denmark; SDU; Novo Nordisk Foundation

**Keywords:** diet modification, obesogens, polychlorinated hydrocarbons, randomized clinical trial, weight gain

## Abstract

**BACKGROUND:**

The purpose was to test the hypothesis that exposures to organochlorine compounds are associated with body weight increases in a dietary intervention study.

**METHODS:**

In the DioGenes trial, adults with obesity who had at first lost at least 8% of their body weight then completed at least 26 weeks on a specific diet. Concentrations of major organochlorine compounds were assessed in plasma samples obtained at study baseline.

**RESULTS:**

A total of 372 participants with complete data were examined for plasma concentrations of major organochlorine compounds. A doubling in total-PCB in plasma was associated with an increase in weight (in kg) at 26 weeks by 0.43 (0.04;0.83), independent of diet group and sex. Associations for most individual organochlorines were in the same direction, though mostly not statistically significant, especially after adjustment. However, *p,p’*-DDE showed opposite effects. Adjustment for exposure to perfluorinated alkyl substances (PFASs) only minimally affected the findings.

**CONCLUSIONS:**

Elevated plasma concentrations of some organochlorine compounds were weakly associated with increased weight gain, although most individual associations did not reach statistical significance after adjustment for PFAS exposure. However, p,p’-DDE concentrations showed a clear association with lowered body weight. Overall, the halogenated pollutants examined are likely to contribute to the obesity pandemic.

**TRIAL REGISTRATION:**

The original RCT is with ClinicalTrials.gov number NCT00390637.

## BACKGROUND

Overweight and obesity have serious adverse impacts on public health internationally, and the prevalence is increasing toward highly worrisome levels [1]. Despite the major focus on lifestyle factors [1], substantial evidence suggests that environmental chemicals likely play a large role in the development of overweight and obesity [2]. In particular, several persistent organic pollutants (POPs) may well act as obesogens and contribute to adipose tissue development [3], especially the polychlorinated biphenyls (PCBs) that exhibit endocrine disrupting properties [4]. Obesogenic properties of PCBs and related organochlorine compounds (OCs) have been documented in experimental animal studies, while some human studies have reported associations with overweight, in particular in highly exposed children [4–7]. Of note, the persistent pesticide DDT and its DDE metabolite have also been linked to obesity [7, 8]. A major concern in regard to the etiology of overweight is the possibility of confounding, especially in regard to concomitant and past exposures. Thus, obesity may be affected by exposure to other environmental toxicants, such as the perfluorinated alkylate substances (PFASs) [9], as suggested by our previous report from the Diogenes clinically controlled study [10]. Experimental toxicology studies suggest impact on important functions, such as energy metabolism, glucose control, and thyroid hormone homeostasis that may result from exposures to PCBs [3] and PFASs [11].

Given the impossibility of conducting clinical trials of toxicant exposures, a feasible alternative is to examine chemical exposures in dietary interventions aimed at inducing and maintaining weight loss [12, 13]. Due to the long biological half-life of several obesogens [11, 14], their concentrations in serum or plasma measured at baseline are likely to remain virtually unchanged throughout the duration of a one-year study duration. Still, weight loss may possibly result in the release of some lipophilic substances, like PCBs, stored in lipid tissues [15]. In studies of obesogens, the maintenance of a weight loss following calorie restriction is a key challenge that can serve as a sensitive outcome [10, 16]. We have benefitted from access to a dietary intervention trial conducted jointly in eight European countries [16], where biobanked plasma from trial participants allowed determination of exposures to environmental chemicals at baseline [10].

## METHODS

### Study design

The present study relies on the Diet, Obesity and Genes (DioGenes) European multicenter trial, conducted in 2006–2008, where we previously measured the baseline plasma-PFAS concentrations and their possible associations with weight change during dietary intervention in trial participants evenly distributed in eight European countries [10]. The DioGenes RCT (ClinicalTrials.gov number NCT00390637) focused on the importance of a slight increase in dietary protein content and lowering of the glycemic index of the carbohydrates for weight control in families with obesity [16]. The adults first underwent a diet of 800 kcal per day for 8 weeks, and those losing at least 8% of their initial body weight qualified for randomization into one of five *ad libitum* diets for 26 weeks, with an optional continuation for another 26 weeks. Between clinical examinations, participants recorded their morning body weight according to a detailed instruction. After the 26-week intervention period, food and instructions were no longer provided, and many chose to discontinue their participation during this period, while 101 remained up to the maximum duration of 26 additional weeks [17].

The five *ad libitum* diets included the following: low-protein and low-glycemic index (GI), low-protein and high GI, high-protein and low GI, high-protein and high GI, or a healthy control diet. The effect of high protein and low glycemic index was found to be additive on weight maintenance, and their combination was successful in limiting weight regain after randomization. While substantial interindividual variability was present, specific diets clearly helped prevent weight regain under the *ad libitum* conditions [12].

#### Exposure assessment

Sufficient amount of plasma from baseline before the initial weight loss (≥ 75 μL) was available from 372 out of the 548 participants who had completed the intervention, i.e., nine participants from our PFAS study had insufficient plasma volume to be included in the present study. Body weight (in kg with one decimal) was available at multiple points after randomization, but almost half of the trial participants dropped out of the study after 26 weeks, and only 101 of those with plasma available remained through the extended period up to 52 weeks.

Baseline plasma-OC concentrations were measured at the University of Southern Denmark, by a sensitive and reliable method based on the principle of isotope dilution, solid-phase extraction and gas chromatography coupled to a triple quadropole mass spectrometer [18]. The concentrations measured were adjusted for total lipid content determined on a kit-based routine analysis on a Konelab 20 Clinical Chemistry Analyzer. The limit of detection (LOD) for the PCBs, *p,p’*-dichlorodiphenyldichloroethylene (*p,p’*-DDE) and hexachlorobenzene (HCB) was 0.03 ng/mL which corresponds to 0.003 μg/g lipid at an average serum-lipid concentration of 10 g/L. All results below the LOD were replaced by LOD/2. The inter- and intra-assay coefficients of variation (CV) were < 13%. The accuracy of the analysis has continuously been controlled through biennial participation in the German-External Quality Assessment Scheme (G-EQUAS), organized by Institute of OutPatient Clinic for Occupational, Social and Environmental Medicine of the University of Erlangen-Nuremberg, Germany. While we have previously examined PFAS concentrations in the present trial, OCs are now included, as they may also contribute to endocrine disruption, as documented in a large group of U.S. women at background exposures [19].

### Statistical analysis

We used the same analytical approach as in our previous study [10], though now with focus on the major PCBs (congeners 138, 153 and 180), as well as the dioxin-like congener 118 and ∑PCB (sum of the three major PCBs multiplied by 2.0). We also measured HCB and *p,p’*-DDE concentrations. Serum-OC concentrations are adjusted by the total lipid concentration in the sample. Samples with results below the detection level were assumed to contain 0.015 μg/g lipid.

Descriptive statistics are provided for baseline characteristics and the pollutant distributions across the different groups, where all continuous variables were grouped based on their tertiles. Median and interquantile range are shown for each of the PCBs, *p,p*’-DDE and HCB.

The association between plasma-OC concentrations and changes in body weight during the weight maintenance phase was examined by linear mixed regression models, as before [10]. The main model considered all weights measured across the study duration and assumed that the weight changes in participants who dropped out of the study had followed the same course. We used random effects to allow results from the same subject to be correlated.

A random-effects model for each of the OCs was fitted. As previously described for the DioGenes trial [12], covariates for adjustment included baseline age, sex, weight loss achieved during the initial low-calory diet, maintenance diet group, baseline body-mass index (BMI), type of center (shop or instruction) and family type (single-parent or both parents). A time-varying effect by the type of maintenance diet, sex, and plasma-OC concentration was assumed by introducing an interaction term with the number of weeks from randomization. Time (weeks) since randomization was added in the model in the form of a cubic spline, and a likelihood ratio test was performed to identify potential interactions with time. A random intercept was considered together with random slopes for linear and quadratic terms of weeks to account for the correlation between weight measurements from the same individual. The model was implemented for PCB congeners separately, for the three PCBs together (∑PCB), *p,p’*-DDE and HCB. Each OC concentration entered the model after logarithmic transformation (base 2), and the estimated regression coefficients are expressed as the difference in body weight for a doubling in the plasma concentration. Results are shown also when adjusting for the PFAS concentrations (PFOA, PFNA, PFDA, PFHxS, and total PFOS) at week 26 and 52 from randomization.

Given that previous studies had shown that body weight changes might be affected by PFAS exposures at baseline, a quantile g-computation analysis was implemented to assess the association between weight change and the mixture of PFAS exposures [20], defined here as a weighted sum of the PFAS concentrations, while adjusting for PCBs and other OCs. This method fits a marginal structural model with all exposures and assesses the effect of the sum of regression coefficients for all concentrations providing the estimated change in body weight for a doubling of all PFAS concentrations. This model was constructed as described above, with the same covariate adjustments and random effects.

In addition, a sensitivity analysis was conducted using a multiple regression model considering only weight gain information at week 26. The model included adjustment for baseline age, sex, weight loss achieved during the initial low-calory diet, maintenance diet group, BMI, type of center and family type. In this model we re-estimated the exposure effects and compared them to the random effects model. Further, we checked the assumption of log-linear effects by fitting more flexible spline models. Finally, we explored possible interactions between total PCB exposure and sex, age, initial weight loss and diet group.

## RESULTS

### Exposure characteristics

The main characteristics of the 372 participants included are shown in Table 1, together with medians and interquartile ranges of the PCB concentrations in each subgroup. In general, a slightly higher concentrations were observed in males and in older subjects. Similar distributions were observed across diet intervention groups, while *p,p’*-DDE showed a negative association with the total PFAS concentration. An opposite trend was found for the PCBs. This tendency was also confirmed by the inter-correlations between the contaminants (Supplement Fig. 1). Here, a negative correlation was observed between HCB and *p,p’*-DDE with the PFASs. Moreover, the three major PCBs (138, 153 and 180) showed the closest correlation with PFASs, though with a weaker association for PCB 118.

### Relations to weight changes

Serum-PCB results were evaluated both in regard to the estimated ∑PCB and the four individual PCB congeners as well as the two other major OCs (Table 2). The estimated weight change for a doubling of the concentration is given at week 26 (Table 2). An increase in weight change was observed for PCBs 138-153-180 by 0.46 kg (95% C.I.: 0.03;0.89), 0.32 kg (95% C.I.: −0.01;0.65) and 0.38 kg (95% C.I.: 0.05;0.72), respectively. Notably, *p,p’*-DDE showed associations with weight change in the opposite direction (at week 26, −0.66 kg), i.e., elevated DDE concentrations seemed to protect against weight increase in this intervention study. For HCB and PCB118, associations with weight change at week 26 were close to zero and far from statistically significant. Similar results were found at week 52 (Table 3), where wider confidence intervals were observed because of the reduced sample size. Interaction tests for possible modification of the OC effect by diet intervention, age and initial weight loss were far from reaching statistical significance (p = 0.37, p = 0.69 and p = 0.64, respectively). However, the association between the total PCB and weight change at week 26 significantly depended on sex (p = 0.017) and seemed to be much stronger among females (0.67 kg, 95% C.I.: 0.18, 1.15; N = 262) than among males (−0.38 kg, 95% C.I.: −1.14,0.37; N = 110).

**Figure 1:**
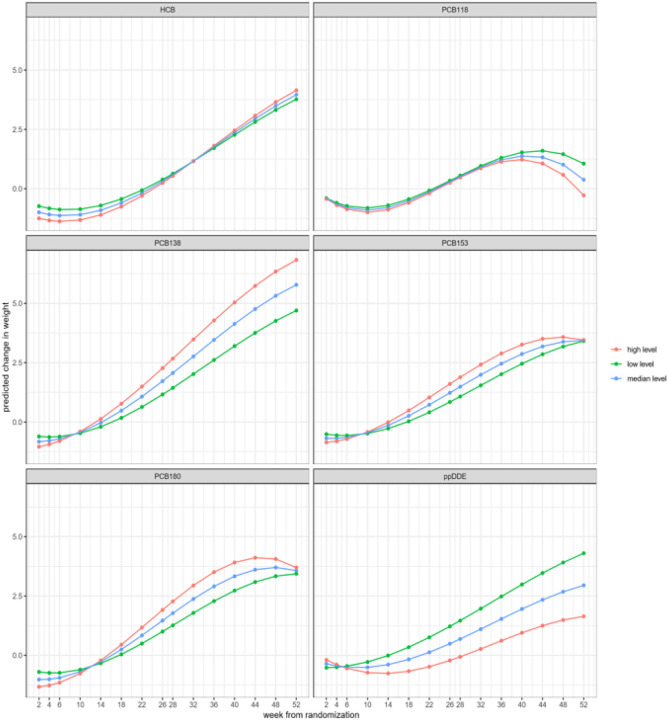
Predictions of weight change over time obtained by the random-effects model where the exposure variable was fixed at the 25th (low level), 50th (median level) and 75th (high level) percentile of its empirical distribution. The individual-level predictions were then averaged across the sample to obtain the marginal population-level estimates of weight change over time.

Figure 1 shows the predicted weight change trajectories at different levels of exposure for each of the major OCs. The predictions were obtained in a random-effects model, as described above, where the exposure variable was fixed at the 25th (low level), 50th (median level) and 75th (high level) percentile of its empirical distribution. Here, the reversed effect of *p,p’*-DDE is illustrated by the fact that the high exposure level has the smallest increase of weight, and the difference seems to increase with time. Most PCBs show the opposite trend, with the largest weight increase at the low exposure level. The exposure effects at week 26 estimated in the random effects model were similar to those obtained in the multiple regression model based only on weight gain data at week 26 (Supplement Table 1). A check for the log-linearity assumption was implemented for each exposure. When using a more flexible spline model, *p,p*’-DDE showed a null effect at lower concentrations, and a decrease in weight change at higher concentrations (Supplement Fig. 2).

Although the correlation between PFAS concentrations and the lipid-based OCs was weak, we repeated the calculation after adjustment of the OC results for PFAS. Neither set of results was strongly affected by this adjustment (Tables 2 and 3), but the *p,p’*-DDE effect became somewhat smaller and statistically less significant.

For comparison with our previous analyses of weight change in relation to PFAS exposure, a g-computation model was used to assess the mixture effect on weight change for the PFASs, while adjusting for the total PCBs and *p,p’*-DDE. By 26 weeks, the weight increased by 1.29 kg (95% C.I. 0.42, 2.15) for a doubling of total PFAS concentrations, while there was a less clear increase by 0.69 kg (95% C.I. −1.12, 2.51) by week 52.

## DISCUSSION

The present study relied on a randomized weight maintenance intervention study in eight European countries, where plasma concentrations of chlorinated hydrocarbons at baseline have now been determined. The main finding of this study is that elevated exposures of major PCBs were associated with a slight body weight increase after the initial weight loss, independently of the diet assignment. Due to the weak association with PFAS exposures and the similarly in OC exposures in the randomized diet groups, the original findings of the DioGenes trial [12] are therefore not challenged, although other such trials may not be as fortunate.

Certain air pollutants and industrial chemicals have been identified as likely obesogens [2–4], and among the most persistent and bioactive substances are major OCs, especially the PCBs [3, 4, 21]. These substances occur widely in the environment and are consistently found in human blood. Due to the lipophilicity of most OCs and differences in sources and in toxicokinetic fate, serum-OC concentrations seem not to correlate well with other potential obesogen markers that may be less lipophilic [3, 22], thus making confounding less likely.

The present study relied on existing data from a dietary trial study carried out in eight European countries. We adjusted for the same set of covariates as previously identified [12], and we allowed a flexible, time-varying effect of covariates. We employed a random-effects model with a random intercept and random slopes for linear and quadratic terms of weeks. Although the statistical modeling differs somewhat from the original report on the dietary trial [12], the approaches are comparable and efficiently explore the goals of the analysis. We previously reported that differences in PFAS exposure were related to weight changes that were as large as or greater than those associated with the trial diets [10]. In the present study, we observed additional and independent effects of PCB exposures, while DDE exposure seemed to protect against weight gain.

Other human studies have linked elevated OC exposure to metabolic abnormalities, such as increased risk of type 2 diabetes, elevated serum-lipids, and thyroid dysfunction [21, 23]. Given the experimental support [8], the present study adds to the evidence that OCs may contribute to obesogenic effects in humans. Still, *p,p’*-DDE has only been considered a “presumed” obesogen [8].

Participants in the present study were overweight and exposed to background levels of OCs, as average serum concentrations in DioGenes are similar to those reported from other European countries during the same time period [24–26]. Thus, as possible contributors to the obesity pandemic [27], our findings should raise attention to potentials for preventing environmental chemical exposures, including OCs such as the PCBs.

While several studies have suggested that *p,p’*-DDE may have obesogenic effects similar to the PCBs, the evidence mainly relates to weight gain in infants and children, who have been exposed via human milk during a period of rapid growth [7, 21]. Some studies have measured OCs, including DDT and DDE, though without a major focus on DDE. If considered, effects were apparently weak [21, 23, 28]. Given that *p,p’*-DDE is a presumed obesogen, perhaps our results are surprising for a randomized clinical trial, while most of the previous reports are based on cross-sectional studies. Thus, although our findings suggest that this OC does interact with the development of obesity, the DDE exposure results in a lower gain of body weight than is otherwise associated with other contaminants considered obesogenic. Given the strong findings and apparent lack of confounding, the mechanisms and pathways deserve further exploration. One possibility is that elevated serum concentrations of *p,p’*-DDE at the study baseline may reflect elevated exposures to the parent DDT pesticide in the past, and that the weight changes linked to the current concentration of the metabolite may be due, in part, to benefits of DDT breakdown. However, this possibility is speculative at this point and would need prospective evidence for clarification. In addition, the possible association with PFAS exposure may require further attention.

This study comprised a somewhat heterogenous study population recruited from eight European countries based on elevated body weight. Although randomized in parallel in the participation centers, the participants may not necessarily be representative of the general populations. Still, contemporary OC exposures in European countries [3, 4, 6] were comparable to those measured in this study, thus speaking against important selection bias. PFAS exposures appeared as major determinants of weight gain [10], and the lack of clear associations with OC concentrations suggest that confounding is unlikely to have affected our previously reported findings. Unmeasured confounders of possible importance in this observational study could include education or social factors that may potentially be related both to higher obesogen exposures and to lower achievement in dietary weight loss and maintenance programs. While this prospective dietary trial carefully recorded individual changes in body weight at well-defined ad-libitum diets, underlying metabolic changes were not explored. As experimental studies of contaminant exposures in humans are not appropriate, the present trial, in conjunction with related experimental and epidemiological evidence, offers support to a hypothesis of PCB obesogenicity. Given the severity of the current pandemic of overweight and obesity, where over half of the adult population in 2050 is forecasted to be affected [1], the impact of obesogens needs to be taken into serious consideration.

## CONCLUSIONS

In this study of Europeans with obesity, elevated plasma-OC concentrations therefore predicted increased weight gain after an initial weight loss, notwithstanding the diet group that the subjects were assigned to. The results suggest that OC exposure may affect weight change among people with obesity in weight loss programs. These pollutants deserve attention in public health efforts to control the obesity pandemic.

## Supplementary Material

This is a list of supplementary files associated with this preprint. Click to download.

- Supplementalmaterials.docx

## Figures and Tables

**Table 1:** Subject characteristics at baseline and exposure distributions (median and interquartile range).

	PCB138	PCB153	PCB118	PCB180	HCB	pp-DDE
**Gender** Male(n = 110)	0.064 [0.039, 0.098]	0.089 [0.055, 0.141]	0.009 [0.006, 0.014]	0.069 [0.041, 0.124]	0.031 [0.020, 0.066]	0.236 [0.133, 0.438]
Female (n = 262)	0.037 [0.023, 0.066]	0.05 [0.029, 0.092]	0.008 [0.002, 0.012]	0.037 [0.021, 0.068]	0.027 [0.016, 0.073]	0.241 [0.108, 0.518]
p-value	< 0.001	< 0.001	0.134	< 0.001	0.337	0.96
**Age at baseline** [24,39] (n = 125)	0.036 [0.020, 0.056]	0.045 [0.024, 0.075]	0.007 [0.002, 0.010]	0.031 [0.014, 0.067]	0.023 [0.016, 0.046]	0.218 [0.103, 0.428]
(39,45] (n = 131)	0.05 [0.032, 0.074]	0.073 [0.041, 0.103]	0.009 [0.006, 0.014]	0.054 [0.033, 0.086]	0.031 [0.018, 0.087]	0.197 [0.106, 0.453]
(45,63] (n = 116)	0.046 [0.028, 0.091]	0.065 [0.035, 0.132]	0.009 [0.006, 0.014]	0.056 [0.027, 0.116]	0.029 [0.017, 0.086]	0.298 [0.129, 0.724]
p-value	< 0.001	< 0.001	< 0.001	< 0.001	0.04	0.022
**Randomized diet** Healthy diet (n = 79)	0.041 [0.025, 0.082]	0.058 [0.035, 0.103]	0.009 [0.005, 0.013]	0.043 [0.026, 0.075]	0.028 [0.018, 0.053]	0.212 [0.102, 0.517]
High protein/Low GI (n = 84)	0.046 [0.025, 0.071]	0.055 [0.034, 0.109]	0.008 [0.002, 0.012]	0.05 [0.025, 0.107]	0.031 [0.014, 0.112]	0.255 [0.111, 0.486]
High protein/High GI (n = 74)	0.046 [0.026, 0.067]	0.058 [0.035, 0.109]	0.009 [0.003, 0.014]	0.048 [0.025, 0.108]	0.027 [0.016, 0.088]	0.305 [0.118, 0.571]
Low protein/Low GI (n = 77)	0.041 [0.026, 0.083]	0.056 [0.029, 0.088]	0.007 [0.005, 0.011]	0.043 [0.025, 0.075]	0.028 [0.017, 0.050]	0.211 [0.124, 0.445]
Low protein/High GI(n = 58)	0.051 [0.029, 0.074]	0.076 [0.043, 0.116]	0.009 [0.005, 0.014]	0.053 [0.029, 0.112]	0.029 [0.018, 0.067]	0.186 [0.114, 0.480]
p-value	0.79	0.403	0.528	0.689	0.994	0.763
**Weight loss during LCD** [5.6,9.2] (n = 123)	0.043 [0.024, 0.073]	0.055 [0.036, 0.103]	0.008 [0.005, 0.012]	0.048 [0.026, 0.107]	0.029 [0.016, 0.116]	0.24 [0.101, 0.636]
(9.2,11.8] (n = 123)	0.047 [0.030, 0.073]	0.065 [0.036, 0.107]	0.01 [0.006, 0.014]	0.045 [0.029, 0.079]	0.028 [0.018, 0.058]	0.227 [0.115, 0.411]
(11.8,28.3] (n = 126)	0.04 [0.023, 0.074]	0.059 [0.029, 0.108]	0.008 [0.002, 0.012]	0.043 [0.021, 0.086]	0.027 [0.016, 0.048]	0.25 [0.138, 0.511]
p-value	0.365	0.715	0.274	0.43	0.351	0.462
**BMI** [26.6,31.4] (n = 123)	0.046 [0.027, 0.074]	0.064 [0.038, 0.108]	0.009 [0.006, 0.013]	0.053 [0.033, 0.095]	0.025 [0.016, 0.064]	0.18 [0.085, 0.361]
(31.4,35.7] (n = 123)	0.05 [0.031, 0.087]	0.07 [0.038, 0.121]	0.008 [0.006, 0.013]	0.054 [0.030, 0.111]	0.032 [0.018, 0.071]	0.251 [0.128, 0.565]
(35.7,45.9] (n = 126)	0.033 [0.022, 0.063]	0.044 [0.024, 0.091]	0.008 [0.002, 0.012]	0.03 [0.017, 0.071]	0.029 [0.017, 0.074]	0.294 [0.138, 0.555]
p-value	< 0.001	0.001	0.226	< 0.001	0.333	0.003
**Weight at start** [66.6,89.1] (n = 123)	0.041 [0.025, 0.074]	0.056 [0.035, 0.096]	0.008 [0.005, 0.012]	0.047 [0.027, 0.101]	0.025 [0.015, 0.099]	0.182 [0.096, 0.500]
(89.1,104] (n = 123)	0.048 [0.029, 0.073]	0.068 [0.036, 0.108]	0.009 [0.006, 0.014]	0.055 [0.028, 0.099]	0.034 [0.018, 0.071]	0.261 [0.120, 0.568]
(104,159] (n = 126)	0.042 [0.025, 0.073]	0.056 [0.026, 0.099]	0.007 [0.002, 0.012]	0.039 [0.021, 0.076]	0.028 [0.018, 0.054]	0.249 [0.140, 0.495]
p-value	0.366	0.196	0.096	0.222	0.504	0.269
**Waist circumference** [73.8,100] (n = 122)	0.04 [0.025, 0.080]	0.057 [0.033, 0.112]	0.008 [0.002, 0.012]	0.047 [0.027, 0.114]	0.026 [0.015, 0.117]	0.19 [0.078, 0.527]
(100,110] (n = 123)	0.054 [0.028, 0.080]	0.077 [0.037, 0.108]	0.01 [0.007, 0.014]	0.056 [0.028, 0.092]	0.028 [0.017, 0.047]	0.24 [0.131, 0.466]
(110,153] (n = 123)	0.04 [0.026, 0.071]	0.055 [0.032, 0.098]	0.007 [0.002, 0.012]	0.037 [0.023, 0.075]	0.029 [0.018, 0.060]	0.251 [0.134, 0.487]
Missing (n = 4)	0.033 [0.023, 0.043]	0.034 [0.024, 0.045]	0.012 [0.009, 0.026]	0.02 [0.017, 0.025]	0.027 [0.023, 0.030]	1.585 [1.229, 1.984]
p-value	0.182	0.077	0.01	0.093	0.976	0.178
**Total PFAS** low (n = 123)	0.039 [0.020, 0.073]	0.052 [0.020, 0.104]	0.007 [0.002, 0.012]	0.041 [0.016, 0.114]	0.032 [0.016, 0.141]	0.321 [0.161, 0.678]
middle (n = 123)	0.046 [0.026, 0.085]	0.058 [0.035, 0.108]	0.009 [0.006, 0.014]	0.045 [0.026, 0.120]	0.032 [0.017, 0.106]	0.282 [0.111, 0.572]
high (n = 126)	0.047 [0.031, 0.073]	0.065 [0.043, 0.102]	0.009 [0.006, 0.012]	0.049 [0.030, 0.071]	0.026 [0.018, 0.037]	0.163 [0.090, 0.274]
p-value	0.093	0.1	0.019	0.429	0.011	< 0.001

**Table 2:** Estimated change in weight gain (kg) from randomization to **week 26** for a doubling of individual serum-PCB concentrations. The change in weight gain is estimated by random-effects models adjusted for baseline age, sex, initial weight loss, maintenance diet group, baseline body-mass index (BMI), type of center and family type, with and without adjustment for PFAS exposure.

	Unadjusted for PFAS		Adjusted for PFAS	
	Estimate (95% C.I.)	p-value	Estimate (95% C.I.)	p-value
PCB138	0.46 (0.03;0.89)	0.036	0.45 (0.03;0.87)	0.037
PCB153	0.32 (−0.01;0.65)	0.058	0.25 (−0.08;0.58)	0.135
PCB180	0.38 (0.05;0.72)	0.026	0.31 (−0.01;0.64)	0.061
PCB118	0.36 (−0.02;0.73)	0.064	0.33 (−0.04;0.71)	0.081
∑PCB	0.43 (0.04;0.83)	0.032	0.37 (−0.02;0.76)	0.063
*p,p’*-DDE	−0.66 (−1.00;−0.32)	< 0.001	−0.46 (−0.82;−0.10)	0.013
HCB	−0.05 (−0.35;0.24)	0.713	0.11 (−0.18;0.40)	0.461

**Table 3:** Estimated change in weight gain (kg) from randomization to **week 52** for a doubling of individual serum-PCB concentrations. The change in weight gain is estimated by random-effects model adjusted for baseline age, sex, initial weight loss, maintenance diet group, baseline body-mass index (BMI), type of center and family type, with and without adjustment for PFAS exposure.

	PFAS unadjusted		PFAS adjusted	
	Estimate (95% C.I.)	p-value	Estimate (95% C.I.)	p-value
PCB138	0.89 (−0.10;1.88)	0.078	1.04 (0.02;2.06)	0.045
PCB153	−0.01 (−0.80;0.78)	0.981	0.01 (−0.80;0.83)	0.971
PCB180	0.35 (−0.53;1.23)	0.440	0.42 (−0.49;1.33)	0.366
PCB118	0.14 (−0.59;0.87)	0.701	0.22 (−0.55;0.98)	0.581
tot_PCB	0.26 (−0.78;1.30)	0.621	0.40 (−0.70;1.50)	0.474
*p,p’*-DDE	−1.23 (−1.97;−0.49)	0.001	−0.79 (−1.58;−0.01)	0.048
HCB	0.14 (−0.59;0.87)	0.700	0.44 (−0.32;1.20)	0.253

## Data Availability

Information on DioGenes procedures and data availability are available at https://cordis.europa.eu/project/id/513946/reporting
